# Exploring the Effects of Pemivibart Monoclonal Antibody Infusion in Long COVID: A Case Series Offering Initial Clinical Insights

**DOI:** 10.7759/cureus.96246

**Published:** 2025-11-06

**Authors:** John M Baratta, Kayla A Jensen, Cameron Cobb

**Affiliations:** 1 Department of Physical Medicine and Rehabilitation, University of North Carolina School of Medicine, Chapel Hill, USA

**Keywords:** brain fog, chronic fatigue, long covid, monoclonal antibody covid-19, post-acute sequelae of sars-cov-2 infection (pasc)

## Abstract

Long COVID, or post-COVID conditions (PCC), affects a substantial proportion of adults and is characterized by persistent symptoms such as fatigue, postexertional malaise (PEM), cognitive dysfunction, and dysautonomia. At present, no Food and Drug Administration-approved therapies exist. Pemivibart (Pemgarda), a monoclonal antibody (mAb) authorized under Emergency Use Authorization (EUA) for preexposure prophylaxis in immunocompromised individuals, has not been studied for PCC but may theoretically facilitate clearance of viral antigens.

We report three individuals with longstanding, debilitating PCC who received pemivibart infusion under EUA. Each experienced rapid symptomatic improvement, most notably in fatigue with PEM, dysautonomia, and cognitive dysfunction. One patient achieved resolution of her most limiting symptoms within a week and sustained functional recovery following repeat infusion. Another demonstrated significant gains across several symptom domains and was able to resume prior physical activities, although benefits waned after two to three weeks. A third with autoimmune comorbidities reported dramatic improvement in energy and cognition, and continued to feel well at six weeks.

The rationale for this case series stems from emerging hypotheses that persistent viral antigens may contribute to the pathophysiology of Long COVID and that neutralizing antibodies could facilitate antigen clearance and symptom resolution. In the three cases, pemivibart infusion was temporally associated with rapid and meaningful improvement in fatigue associated with PEM, cognitive dysfunction, and dysautonomia, symptom clusters that often drive functional impairment in PCC. While the magnitude and duration of benefit varied among individuals, the consistent pattern of improvement supports further exploration of mAb therapy as a potential disease-modifying approach. These preliminary findings highlight the need for controlled, biomarker-guided studies to determine efficacy, durability, and patient subgroups most likely to respond. Pemivibart remains authorized only for preexposure prophylaxis in immunocompromised individuals and is not approved for the treatment of PCC.

## Introduction

Post-COVID conditions (PCC), also termed Long COVID or post-acute sequelae of SARS-CoV-2 infection, are a group of chronic health conditions characterized by persistent, often multisystem symptoms that impair function and quality of life [1]. Cross-sectional surveys indicate that approximately 18% of the population has experienced PCC and, as of late 2024, that the condition continued to affect approximately 5% of U.S. adults [2]. Common symptom domains include fatigue, postexertional malaise (PEM), cognitive dysfunction, dysautonomia, dyspnea, and cardiovascular complaints, driving increased healthcare utilization and lost productivity [3,4]. Despite its substantial burden, there are no treatments approved by the U.S. Food and Drug Administration (FDA) specifically for PCC; care remains supportive and symptom-targeted.

Converging evidence implicates immune dysregulation and viral antigen persistence among leading mechanistic hypotheses. Reviews and mechanistic studies describe chronic immune activation, endothelial dysfunction, and neuroinflammation in PCC [3,5,6]. These reviews highlight evidence of sustained cytokine elevation, altered T- and B-cell phenotypes, microglial activation, and autoantibody production that may perpetuate systemic and neurologic symptoms long after acute infection. Multiple cohorts using ultrasensitive assays have detected circulating SARS-CoV-2 antigens, often the S1 spike protein, months after acute infection [7,8]. These findings suggest ongoing low-level viral antigen production or delayed clearance rather than simple residual debris, implying the presence of a biologically active viral reservoir capable of sustaining immune activation and inflammatory signaling long after the initial illness. Tissue-based studies have similarly identified viral RNA or protein in gut, lymphoid, and cardiovascular compartments well beyond initial infection [9]. While the causal role of these findings remains debated, they suggest that antiviral and immunologic strategies warrant investigation in patients with PCC.

Monoclonal antibodies (mAbs) against SARS-CoV-2 were initially developed for the prevention or early treatment of acute COVID-19. Pemivibart (Pemgarda) is currently authorized by the FDA under Emergency Use Authorization (EUA) for preexposure prophylaxis in certain immunocompromised individuals; it is not FDA-approved and is not authorized for treatment of acute COVID-19 or PCC [10]. Nevertheless, by neutralizing virus or viral fragments, thereby potentially facilitating clearance of residual antigen, mAbs could plausibly benefit a subset of patients with PCC driven by viral persistence. Early clinical signals are limited, but isolated case reports and small series describe symptomatic improvement after anti-SARS-CoV-2 mAb administration, and a randomized phase II trial of another mAb (sipavibart) is underway in PCC [11,12].

In this context, we present a case series describing clinical characteristics, treatment course, and patient-reported outcomes following pemivibart administration in individuals with Long COVID. Pemivibart, an mAb authorized under EUA for preexposure prophylaxis in immunocompromised individuals, targets the SARS-CoV-2 spike protein and may facilitate clearance of persistent viral antigens that have been implicated in PCC pathophysiology. Given emerging evidence of viral antigen persistence and immune dysregulation in Long COVID, exploring the potential therapeutic impact of neutralizing antibodies is a biologically plausible yet untested strategy. This report is intended as hypothesis-generating only. All administrations were performed under EUA, and use of pemivibart for Long COVID represents an off-label application outside of the current FDA authorization.

## Case presentation

Case 1

In April 2024, a 70-year-old woman with a history of numerous allergies, including an allergic response to the Moderna COVID-19 vaccine (Moderna, Inc., Cambridge, Massachusetts), developed severe COVID-19 with significant dyspnea. After resolution of the initial infection, she experienced ongoing breathlessness, along with PEM, dysautonomia, digestive issues, syncopal episodes, and intermittent cognitive fog. Workup with her pulmonologist, including pulmonary function testing, was unrevealing, other than mildly reduced diffusing capacity (Table 1). Symptoms were felt to be related to Long COVID.

**Table 1:** Pulmonary function testing for Case 1 FVC: forced vital capacity; FEV₁: forced expiratory volume in one second; FEV₁/FVC: ratio of forced expiratory volume in one second to forced vital capacity; TLC: total lung capacity; RV: residual volume; DLCO: diffusing capacity for carbon monoxide; DL/VA: diffusing capacity per unit alveolar volume; pre: prebronchodilator measurement; post: postbronchodilator measurement; % pred: percent predicted

Parameter	Pretest (L/% pred)	Posttest (L/% pred)
FVC	3.35 L (95%)	3.25 L (92%)
FEV₁	2.63 L (98%)	2.74 L
FEV₁/FVC	78%	84%
TLC	6.13 L (108%)	-
RV (% Pred)	108%	-
DLCO (uncorrected)	16.25 mL/minute/mmHg (73%)	-
DL/VA (% Pred)	77%	-

Her symptoms progressively worsened over the next few months, with PEM becoming her most debilitating symptom. Before her initial infection, she reported being very physically active, regularly running 3 miles/day. Following COVID-19 and the development of PCC, she was unable to participate in even mild household activities due to severe "crashes” characterized by flaring of PCC symptoms, particularly fatigue. Additionally, she reported experiencing palpitations nearly constantly and developed syncopal episodes on multiple occasions. Diagnostic evaluation, including basic blood work, inflammatory markers, and rheumatologic screening, was unremarkable. She attempted various methods for symptomatic management, both pharmacologic and nonpharmacologic, including low-dose naltrexone, N-acetylcysteine, physical/occupational therapy, breathwork, yoga, and increased salt intake, but none provided durable relief for the symptom constellation.

Due to a history of severe COVID-19 with an inability to take vaccination, she was provided pemivibart infusion for preexposure prophylaxis in February 2025. Over the subsequent several days, the patient reported a dramatic improvement in numerous PCC symptoms. Brain fog and dysautonomia resolved within three days of the infusion, and her PEM resolved within one week. At follow-up two months later, she continued to report a complete resolution of PEM, dysautonomia, and brain fog, and was able to engage in moderate physical activity (weightlifting, tai chi, running) and resume writing a book without issue. In early April, she experienced two episodes of near syncope and palpitations similar to those she experienced before the infusion. The patient received a second pemivibart infusion in July 2025. Within two weeks, she reported further improvement to the PCC, with her cardiologist noting that dysautonomia symptoms "remarkably improved after receiving an infusion of mAbs.”

Case 2

In August 2021, a 63-year-old man developed COVID-19 with symptoms of fever, cough, dyspnea, myalgias, arthralgias, anosmia, and dysgeusia. He was treated with oral steroids and azithromycin in an outpatient setting. After the initial infection, he experienced a myriad of symptoms, including chronic fatigue, PEM, brain fog, dyspnea, arthralgias and muscle pain, paresthesias, headaches, muscle twitching, and depression/anxiety exacerbation. Over the following years, medical evaluation was suggestive of psoriatic arthritis, and his rheumatologist started him on methotrexate.

While his general symptom constellation waxed and waned, it progressively worsened overall, significantly decreasing his function and quality of life. His most bothersome symptoms were fatigue with PEM, generalized pain, occipital headaches, and brain fog. During his postexertional crashes, he experienced daily low-grade fevers, muscle twitching, tremors, headaches, and debilitating fatigue and brain fog. These symptoms caused him to reduce his activity significantly; before the infection, he was very physically active, completing projects in the yard and around the house, but now he was so fatigued that he needed to sit for most of the day and required assistance with instrumental activities of daily living. He acknowledged that this also took a toll on him emotionally, stating he was “half the man (he) used to be.” Medical treatment included low-dose naltrexone and bupropion, both of which were ineffective.

Due to immunosuppression in the setting of medical treatment of psoriatic arthritis, he was provided pemivibart infusion for preexposure prophylaxis in April 2025. The patient noted substantial improvement in his PCC shortly after the infusion. In the first week after the infusion, he did not experience any postexertional crashes and was able to resume some of his old activities, including wood chopping. Patient-Reported Outcomes Measurement Information System-29 (PROMIS-29) scoring performed pre- and postinfusion indicated improvement across several domains, including physical function, anxiety/depression, fatigue, sleep, social function, and pain (Table 2). The patient noted that his benefits persisted for 2.5 weeks, until the fatigue, headaches, fevers, and joint pain returned. He expressed eagerness for repeat infusion in the future.

**Table 2:** Pre- and postinfusion PROMIS-29 T-scores for Case 2 PROMIS-29: Patient-Reported Outcomes Measurement Information System-29

PROMIS-29 domain	Preinfusion T-score	Postinfusion T-score	Interpretation
Physical function	35.6	43.4	Moved from well below average to closer to normal physical functioning
Anxiety	73.3	51.2	Marked reduction in anxiety symptoms, from severe to average
Depression	69.4	49	Significant reduction in depressive symptoms, from moderate/severe to normal
Fatigue	71.6	46	Large drop in fatigue, from very high to below average
Sleep disturbance	63.8	48.4	Sleep problems improved from moderately elevated to within a normal range
Social roles	37.3	51.9	Shift from impaired to average social functioning
Pain interference	66.6	53.9	Substantial reduction in the pain's impact on daily life

Case 3

In May 2021, a 57-year-old woman with a complex medical history, including asthma, insomnia, chronic inflammatory demyelinating polyneuropathy, and Sjogren’s syndrome, developed COVID-19. During the infection, she experienced worsening lower extremity numbness, and she was hospitalized for a persistent sore throat with fevers for a month. She completed the Pfizer COVID-19 vaccine course in September 2021, after which she experienced gastroparesis with irritable bowel syndrome symptoms.

Long COVID symptoms, which developed subsequent to the initial illness, included severe fatigue and PEM, exertional dyspnea, dysautonomia, sleep disturbances, chest pain, brain fog, and frequent migraines. Over the following years, she attempted various medication treatments for the condition including ivermectin, along with multiple supplements such as alpha lipoic acid, bromelain, dandelion, ginger root, magnesium glycinate, N-acetylcysteine, nattokinase, omega-3 fatty acids, a multivitamin, probiotics, resveratrol, selenium, turmeric, vitamin B12, vitamin C, vitamin D3, vitamin E, vitamin K2, and zinc. At the time of presentation to our clinic in June 2025, fatigue with PEM and sleep disturbances continued to significantly disrupt her daily function and overall quality of life.

Due to immunosuppression in the setting of hydroxychloroquine use for autoimmune disease, she was provided with pemivibart infusion for preexposure prophylaxis in August 2025. On the first day after the infusion, she experienced a severe gastrointestinal reaction with generalized body pain, but she subsequently noticed a significant improvement in her symptoms. Over the coming days, she reported feeling “resurrected from the dead,” citing dramatically improved energy and cognitive function. Before the infusion, she had been unable to participate in even mild activities due to PEM, but afterward, she became able to walk on the treadmill and socialize with friends without crashing. Following the infusion, she was noted to have an improvement on the DePaul Symptom Questionnaire-Short Form (DSQ-SF) from 40/40 to 4/40, indicating substantial improvement in symptom constellation (Table 3). At a follow-up visit six weeks after infusion, she noted that her only residual symptom in the weeks following the infusion was ongoing sleep dysregulation. She reported she continued to feel the benefits of the infusion, stating, “I am still doing incredible.”

**Table 3:** DSQ-SF before and after pemivibart infusion DSQ-SF: DePaul Symptom Questionnaire-Short Form

Symptom	Frequency (pre)	Frequency (post)	Δ Frequency	Severity (pre)	Severity (post)	Δ Severity	Interpretation notes
Dead, heavy feeling after starting to exercise	4	1	↓3	4	1	↓3	Substantial improvement in exercise tolerance
The next day soreness or fatigue after nonstrenuous, everyday activities	4	0	↓4	4	0	↓4	Marked improvement in physical fatigue
Mentally tired after the slightest effort	4	1	↓3	4	1	↓3	Improved cognitive stamina; mild residual mental fatigue
Minimum exercise makes you physically tired	4	0	↓4	4	0	↓4	Substantial improvement in exertional tolerance
Physically drained or sick after mild activity	4	0	↓4	4	0	↓4	Marked improvement in postexertional malaise

## Discussion

This case series examined the potential clinical effects of pemivibart, a SARS-CoV-2-neutralizing mAb, in individuals with Long COVID. Three patients with longstanding, debilitating symptoms experienced rapid and meaningful improvement following administration. Across the cases, the most striking benefits were resolution or attenuation of fatigue with PEM, cognitive dysfunction, and dysautonomia, symptom clusters strongly associated with loss of function in PCC [1,2]. Improvements were not only subjectively reported but also reflected in functional outcomes, including resumption of prior physical activities and improved patient-reported outcome scores (e.g., PROMIS-29, DSQ-SF). The temporal relationship between infusion and symptomatic improvement, followed by eventual waning in some cases, suggests a treatment effect rather than spontaneous fluctuation, although placebo and regression-to-the-mean effects cannot be excluded.

The observed improvements are biologically plausible within current mechanistic models of Long COVID. Persistent viral antigens have been identified in plasma and tissue samples months after infection, raising the possibility that ongoing immune activation is perpetuated by residual viral material [7,8]. mAbs against SARS-CoV-2, such as pemivibart, are designed to neutralize circulating virus and promote viral clearance. By reducing antigen burden, mAbs could attenuate downstream immune dysregulation, microvascular dysfunction, or neuroinflammation, processes increasingly implicated in PEM, dysautonomia, and cognitive impairment [6,13].

These cases build upon emerging but limited literature, suggesting the potential benefit of mAb therapy in PCC. In a 2024 case series, Scheppke et al. described individuals with severe, persistent post-COVID symptoms who experienced marked improvement following mAb infusions, including remission of fatigue and cognitive complaints [11]. More recently, a blinded, placebo-controlled trial led by Peluso at the University of California, San Francisco, tested mAb AER002 in 36 patients with Long COVID (Website: Spring 2025 symposium - PolyBio Research Foundation; 2025). While the study results are not yet published, early data presentation indicates that no significant difference in symptom improvement across outcome measures (PROMIS-29, C-reactive protein, interleukin-6) was observed between the antibody and placebo arms, though the treatment was well tolerated and provides critical insight for future trial design.

Divergent findings from prior reports and the present case series highlight both the heterogeneity of response and the need for biomarker-guided approaches to identify the patient subgroups who are most likely to benefit (Website: Spring 2025 symposium - PolyBio Research Foundation; 2025) [11]. In this series, one patient experienced sustained remission of PEM and cognitive symptoms for nearly two months, while another experienced improvements lasting less than three weeks. The third reported dramatic symptomatic relief despite an initial infusion-related adverse reaction. This variability may reflect differences in underlying pathophysiology, comorbid conditions, or host-virus interactions. Identifying biomarkers predictive of response will be critical for guiding future trials and patient selection.

Several limitations are noted in the present report. First, these are uncontrolled case observations without blinded assessments, and natural fluctuation of the illness or placebo effects cannot be excluded. Second, symptom reporting relied on patient accounts, though in two cases, validated patient-reported outcome tools were also employed. Finally, the generalizability of these observations is limited by small sample size, heterogeneity of prior infection, and variability in comorbidities.

Despite these limitations, the consistent and sometimes dramatic symptom improvements across distinct cases highlight mAbs as a potential therapeutic avenue for investigation in PCC. Future research should prioritize randomized, placebo-controlled trials to establish efficacy, optimal dosing intervals, and durability of benefit. Biomarker-driven studies may clarify whether subsets of patients, such as those with evidence of persistent viral antigen, derive particular improvement.

Importantly, pemivibart is currently available only under EUA for preexposure prophylaxis in immunocompromised individuals and is not FDA-approved for the treatment of COVID-19 or Long COVID. Its use in the three cases presented was for preexposure prophylaxis, consistent with EUA guidance, and results should be interpreted with caution. Nevertheless, these cases contribute to a growing body of evidence that targeting viral persistence with neutralizing antibodies may offer relief for some patients with Long COVID, and they underscore the urgent need for systematic investigation of targeted biologic therapies in this population.

## Conclusions

In this case series, three individuals with longstanding, disabling Long COVID experienced rapid and meaningful improvements in PEM, cognitive dysfunction, and dysautonomia following a pemivibart infusion, with benefits ranging from weeks to months and supported by validated patient-reported outcome measures. While placebo effects and natural fluctuations cannot be excluded, the temporal relationship, biological plausibility, and functional gains observed suggest mAb therapy may hold promise for a subset of patients with PCC. These findings underscore the urgent need for controlled, biomarker-guided clinical trials to determine efficacy, durability, and patient selection criteria. Importantly, pemivibart is currently authorized only for preexposure prophylaxis in immunocompromised individuals and is not FDA-approved for the treatment of Long COVID.
